# Material-driven nanoplatforms for precision hydrogen sulfide delivery

**DOI:** 10.1016/j.redox.2025.103909

**Published:** 2025-10-30

**Authors:** Huiting Xu, Yang Liu, Tiandong Chen, Mingxi Li, Fang Yang

**Affiliations:** a State Key Laboratory of Digital Medical Engineering, School of Biological Sciences and Medical Engineering, Southeast University, Nanjing, Jiangsu, 210096, PR China; b School of Biomedical Engineering and Informatics, Nanjing Medical University, Nanjing, 211166, PR China

**Keywords:** Hydrogen sulfide, Gasotransmitter, Dual-concentration effects, Therapeutic nano-delivery

## Abstract

Long regarded as a toxic substance, hydrogen sulfide (H_2_S) is now recognized as an essential gaseous signaling molecule that demonstrates dual modulation capacities in biological regulation and disease progression. Contemporary research delineates the dynamic enzymatic production pathways (mediated by cystathionine β-synthase (CBS), cystathionine γ-lyase (CSE), and 3-mercaptopyruvate sulfurtransferase (3-MST)) alongside spatially organized signaling networks that govern its systemic influence on neuronal integrity, cardiovascular adaptation, and energy metabolism. Within the 10–100 μM range, this gaseous mediator exerts tissue-protective functions through vascular relaxation, suppression of inflammation, and inhibition of cell death. Conversely, imbalanced H_2_S levels—whether insufficient or excessive—correlate with pathological cascades involving neoplastic transformation, synaptic degeneration, and redox imbalance. This analysis systematically examines progress in precision-controlled H_2_S modulation technologies, particularly stimuli-responsive delivery architectures designed to resolve its concentration-dependent paradoxes. Emerging nanoscale delivery systems demonstrate enhanced spatiotemporal resolution in capitalizing on H_2_S's dichotomous bioactivities for managing cerebrovascular pathologies, malignant proliferation, and mitochondrial dysfunction. Current challenges and opportunities are further discussed regarding therapeutic window optimization and biosafety profiling, proposing convergent approaches that integrate material science with systems biology to actualize H_2_S's clinical potential.

## Graphical abstract

**Figure:**
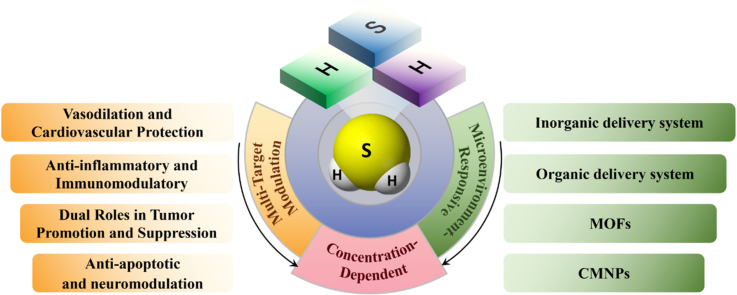

## 1 Introduction

H_2_S, once regarded merely as a toxic industrial gas with a pungent odor, has undergone a paradigm shift in scientific understanding since its identification as the third endogenous gaseous signaling molecule alongside nitric oxide (NO) and carbon monoxide (CO) [[1], [2], [3]]. Historically, H_2_S was first implicated in human health as early as 1713 when Italian physician Bernardino Ramazzini observed its inflammatory effects on sewage workers' eyes, though its chemical identity was later confirmed by Carl Wilhelm Scheele in 1777 [4]. For centuries, H_2_S remained synonymous with toxicity. However, breakthroughs in the late 20th century revealed its essential physiological mediation roles through enzymatic biosynthesis *via* CBS, CSE, and 3-MST [5,6]. These enzymes form a spatially compartmentalized signaling system: intracytoplasmic CBS inhibits glial cell iron death *via* upregulation of GPX4/SLC7A11, reversing the depressive phenotype in rodents; CSE is activated by estrogens to maintain cardiovascular homeostasis *via* the PI3K/Akt pathway, inhibiting myocardial oxidative stress and apoptosis; and mitochondrionally-localized 3-MST catalyzes the generation of peroxisulphide, which can be modified *via* S-peroxidation to against neurodegenerative pathologies. This compartmentalized signaling system enables H_2_S to coordinate diverse physiological processes: cytoplasmic CBS/CSE pathways regulate neuromodulation and vascular dynamics, while mitochondrial 3-MST-derived H_2_S fine-tunes oxidative phosphorylation. Such spatial specialization underscores H_2_S's systemic regulatory capacity across multiple organ systems [7].

At physiological concentrations (10–100 μM), H_2_S exerts multifaceted protective roles, including vasodilation *via* ATP-sensitive potassium (K_ATP_) channel activation, anti-inflammatory effects through NF-κB pathway suppression, as well as anti-apoptotic effects through modulation of the PI3K/Akt channel and pro-survival by intervening in mitochondria [8,9]. Paradoxically, disordered H_2_S contributes to pathology: low concentrations promote tumor angiogenesis and chronic inflammation, whereas excessive H_2_S (>100 μM) induces apoptosis by depleting intracellular cysteine and glutathione (GSH) to increase reactive oxygen species (ROS) expression, underscoring its “double-edged” characteristics [10].

The therapeutic potential of H_2_S depends on precise spatiotemporal control, as its tissue-specific distribution and pleiotropy constitute significant challenges [11]. Recent advances in drug delivery systems aim to mitigate these limitations, thereby propelling H_2_S into the clinic for the treatment of a variety of conditions ranging from cardiovascular ischemia to neurodegenerative disorders, with emerging applications in cancer therapy taking advantage of its concentration-dependent pro-apoptotic effects [[12], [13], [14]]. Despite these advances, challenges remain in optimizing organ-specific delivery and long-term safety, necessitating interdisciplinary innovation to realize the full biomedical potential of H_2_S [15,16].

**Fig. 1:**
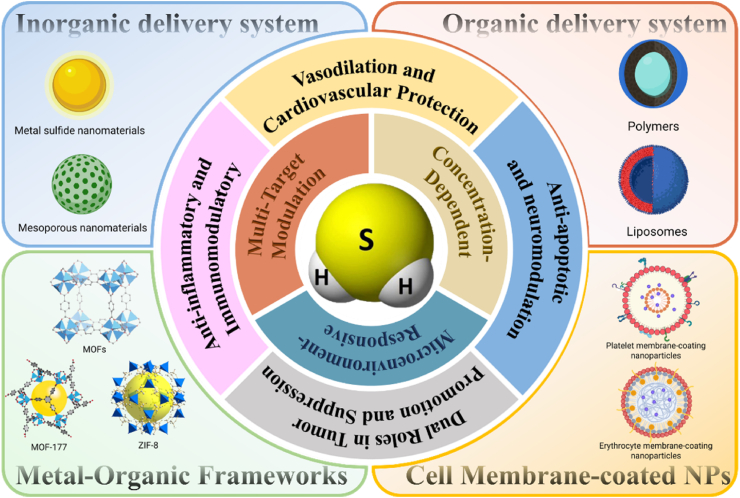
Illustration of H_2_S delivery strategies and their multifaceted biological effects. H_2_S exhibits concentration-dependent and microenvironment-responsive bioactivities, including vasodilatory and cardiovascular protection *via* multi-target modulation, dual anti-apoptotic and neuromodulatory, context-dependent tumor-promoting and tumor-suppressing functions, and anti-inflammatory/immunomodulatory actions. A wide range of nanocarrier platforms, such as inorganic systems, organic systems, metal-organic frameworks, and cell membrane-coated nanoparticles have been developed for H_2_S delivery. This schematic underscores the therapeutic versatility of H_2_S and the need for customized delivery strategies to fully realize its clinical potential.

As shown in Fig. 1, considering the rapid development of gas therapies, this paper systematically evaluates the prospects for the development of H_2_S-related therapies [[17], [18], [19]]. H_2_S gas and H_2_S-producing donors are first described, followed by a brief discussion of H_2_S-mediated signaling cascade pathways involved in therapy [20,21]. Next, existing advanced nanocarrier technologies to ensure the precise release of H_2_S are summarized. While nanotechnology-based drug delivery is a broadly applicable strategy to enhance the pharmacokinetics of various therapeutic agents, its application to H_2_S therapy is not merely an extension of a general approach but is necessitated by the unique pharmacological challenges intrinsic to this gaseous molecule. The paramount challenge lies in the concentration-dependent duality of H_2_S, this narrow therapeutic window demands an unprecedented degree of spatiotemporal control over its delivery, which conventional donors cannot achieve due to their uncontrolled burst release or slow but non-targeted diffusion. Furthermore, H_2_S's high diffusibility, short half-life, and susceptibility to rapid oxidative metabolism *in vivo* further underscore the imperative for advanced delivery systems. Finally, a critical assessment of innovative therapies utilizing the multi-biological effects of H_2_S is presented, looking ahead to possible future biomedical applications.

## 2 H_2_S gas and its traditional donors

### 2.1 H_2_S gas

H_2_S is a simple diatomic gas with a distinctive odor, embodying a paradoxical interplay between physiological regulation and toxic hazard, rooted in its intrinsic physicochemical properties [22,23]. As a small molecule, H_2_S readily diffuses across lipid membranes, enabling rapid interaction with cellular targets without requiring specialized transporters [21]. Its reactivity stems from a unique redox-active sulfur atom, which facilitates direct modification of cysteine residues in proteins through S-sulfhydration, which is a post-translational modification critical for modulating ion channel activity (e.g., K_ATP_ channels) and diverse enzyme functions. For example, S-sulfhydration of CBS at Cys526 enhances its catalytic activity, promoting endogenous H_2_S production in astrocytes and contributing to neuronal redox homeostasis. In contrast, sulfhydration of Caspase-3 inhibits its activation, thereby suppressing the apoptotic cascade. This modification also influences metabolic enzymes such as pyruvate dehydrogenase, where it regulates mitochondrial energy metabolism by switching its catalytic state [[24], [25], [26]]. However, its therapeutic potential is counterbalanced by inherent risks: H_2_S is flammable (explosive range: 4.3–46 % in air) and acutely toxic at elevated concentrations, primarily through mitochondrial cytochrome C oxidase inhibition, which disrupts cellular respiration and induces hypoxia [[27], [28], [29]]. Even at sublethal levels, chronic exposure disrupts neuronal excitability and vascular integrity, underscoring its narrow therapeutic window, complicating its controlled application [21]. Despite these challenges, H_2_S's ability to act as a gaseous signaling molecule—bypassing traditional receptor-ligand interactions—positions it as a unique therapeutic candidate, provided delivery methods (e.g., localized inhalation, nanocarriers) can precisely regulate its spatial and temporal release. Its biological impact thus hinges on a delicate equilibrium between concentration, environmental context, and the molecular machinery it transiently engages [30].

### 2.2 Traditional H_2_S donors

Under pathophysiological conditions characterized by dysregulated endogenous H_2_S systems, exogenous H_2_S supplementation has emerged as a critical therapeutic strategy. In physiological environments (pH = 7.4), H_2_S exists in a pH-dependent equilibrium where ∼80 % dissociates into bisulfide anions (HS^−^) and ∼20 % remains as dissolved gaseous H_2_S [31,32]. This delicate equilibrium underscores the challenges in developing effective exogenous H_2_S donors for clinical translation.

While epidemiological studies associate garlic-derived organosulfur compounds such as Allicin, diallyl sulfide (DAS), diallyl disulfide (DADS), and diallyl trisulfide (DATS) with cancer risk reduction, their therapeutic application as H_2_S donors is limited by unquantifiable release kinetics and non-specific biodistribution [[33], [34], [35]]. In comparison, inorganic sulfide salts (Na_2_S, NaHS) offer precise dosing but exhibit burst-release characteristics incompatible with endogenous H_2_S dynamics. Their rapid hydrolysis (<1 min) and susceptibility to oxidative degradation (HS^−^ → Sx^2−^/SO_3_^2−^) further compromise therapeutic efficacy [36,37].

**Fig. 2:**
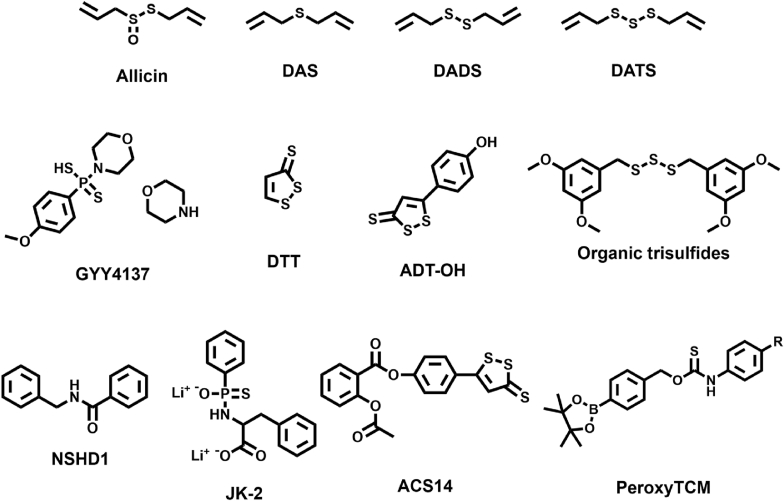
Molecular structures of representative H_2_S donors.

As a result, H_2_S slow-release donors have emerged. Currently, there are many kinds of H_2_S slow-release donors, including hydrolysis-triggered H_2_S donors and thiol-triggered H_2_S donors. Hydrolysis-triggered donors are mainly used in organic synthesis, such as Lawson's reagent (LR) derivatives (GYY4137) and 1,2-dithio-3-thione (DTT) derivatives (ADT/ADT-OH) [[38], [39], [40], [41]]. They can enhance bioavailability by chemical bonding with other functional molecules, such as targeting molecules, potentiating drugs, or reactive linkers. Thiol-triggered donors are controlled-release donors, including organopolysulfides, N-(benzoylthio)benzamide (NSHD1), etc., which release H_2_S *via* thiol exchange and usually contain aromatic groups, releasing more H_2_S than alkyl sulfides [42]. In addition to these, there are also a number of H_2_S donors that are released *via* pathological microenvironmental responsive releases, such as the pH-responsive JK series of donors, protease overexpression-triggered ACS14 [43], and ROS-responsive PeroxyTCM [44]. These biochemical stimulus-responsive H_2_S donors share programmable molecular architectures that allow precise regulation of H_2_S release kinetics, enabling, for example, sustained slow release for chronic inflammation *vs.* stimulus-triggered burst release for tumor therapy. Simultaneously, they enhance tissue-specific accumulation through responsiveness to pathological cues (e.g., acidic pH in tumors, elevated ROS in ischemic tissues) or *via* conjugation with targeting ligands. Despite these advances, challenges persist in minimizing off-target effects and achieving robust tissue-selective delivery with such systems (Fig. 2).

Typically, H_2_S is administered in the form of precursor compounds. However, as summarized in Table 1, its biological effects are strongly concentration-dependent. Concentrations exceeding 100 μM generally exert cytotoxic or pro-apoptotic activity, whereas levels below this threshold tend to induce cytoprotective responses such as vascular remodeling, memory enhancement, and anti-inflammatory effects.

**Table 1:** Summary of recent reported H_2_S pro-drug delivery therapy.

Pro-drug	Concentration	Disease	Effect	Ref.
NaHS	50 μM	Pyemia	The oxidative stress of cardiomyocytes can be alleviated, and the cell vitality can be improved	[45]
NaHS	30 μM	Traumatic brain injury	Activation of the PI3K/Akt pathway inhibits autophagy	[46]
NaHS	50 μM	Hypoxia	Induction of K_ATP_ channel opening, thereby stimulating high stretch-induced atrial natriuretic peptide (ANP) secretion	[47]
GYY4137	1 mM	Colorectal cancer	S-G2/M cycle stagnation was induced	[48]
HA-ADT	200 μM	Breast cancer	Inhibition of PI3K/AKT/mTOR and RAS/RAF/MEK/ERK signaling pathways	[49]

However, in most applications, H_2_S donors require nanocarrier-mediated delivery. The diverse chemical structures and release mechanisms of these donors inherently govern their compatibility with specific nanocarrier strategies. A detailed discussion of advanced micro/nano drug delivery systems for H_2_S delivery has been provided in section 4. Advanced H_2_S micro/nano delivery systems.

## 3 Therapeutic effect mediated by the H_2_S signal pathway

The therapeutic effects of H_2_S are fundamentally determined by its concentration-dependent duality, a core principle governing its signaling behavior. Within physiological concentration ranges (typically 10–100 μmol), H_2_S exerts cytoprotective actions such as vasodilation, anti-inflammatory responses, and anti-apoptotic protection. Deviation from this narrow window, however, leads to markedly divergent outcomes: suboptimal concentrations may fail to confer protection or even exacerbate injury (e.g., impaired angiogenesis during ischemic repair), whereas excessive levels (>100 μmol) provoke cytotoxic effects, including mitochondrial dysfunction and oxidative stress. The following sections 3.13.23.33.4 detail how such concentration gradients direct the biological actions of H_2_S in specific pathological contexts.

### 3.1 Anti-inflammatory and immunomodulatory effects

H_2_S exerts anti-inflammatory and immunomodulatory effects *via* redox-sensitive signaling pathways, with its therapeutic precision dependent on delivery systems that control H_2_S release spatiotemporally [50,51]. Its mechanism involves S-sulfhydration of the p65 subunit to inhibit NF-κB activation, thereby suppressing pro-inflammatory cytokines like TNF-α and IL-6 and alleviating inflammatory cascades in conditions such as inflammatory bowel disease and aortic valve inflammation [[51], [52], [53], [54], [55]]. Additionally, H_2_S drives macrophage polarization from pro-inflammatory M1 to anti-inflammatory M2 phenotypes, remodeling the immune microenvironment. For example, in IL-4-induced macrophages, the H_2_S donor GYY4137 enhances Arg-1 expression without substantially interfering with LPS-induced M1 polarization [56,57].

**Fig. 3:**
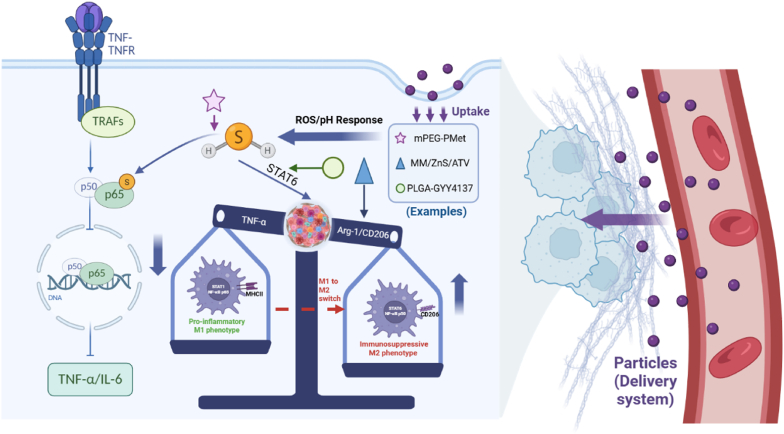
Exogenously delivered H_2_S suppresses inflammation and exerts immunomodulatory effects through dual signaling pathways (Image created by Biorender.com).

The therapeutic precision of H_2_S depends on delivery systems that enable spatiotemporal release to avoid concentration-dependent toxicity and enhance targeting (Fig. 3). For NF-κB inhibition, stimulus-responsive systems are employed: ROS-sensitive mPEG-PMet (loaded with PeroxyTCM) releases H_2_S at ROS-enriched sites (e.g., nerve injuries) to reduce IL-6 [54], while pH-sensitive liposomes (encapsulating JK-series donors) target intestinal inflammation to mitigate mucosal damage [43,55]. In macrophage polarization, targeted delivery is critical. Macrophage membrane-coated MM/ZnS/ATV nanoparticles home to atherosclerotic plaques *via* chemokine receptors and release H_2_S/atorvastatin in acidic microenvironments (pH = 6.5), upregulating M2 markers Arg-1 and CD206 and reducing TNF-α [56]. PLGA-microencapsulated GYY4137 maintains H_2_S within the therapeutic window (50–80 μM), activates STAT6, and enhances Arg-1 expression without disrupting M1 polarization [57,58].

In disease-specific applications, PEG-modified GYY4137 liposomes (circulation half-life extended to 28 h) target the lungs in chronic obstructive pulmonary disease (COPD), releasing H_2_S (>100 μM) to alleviate airway remodeling and reduce mouse airway resistance [59,60]; mesoporous silica nanoparticles (MSNs) delivering 50 μM NaHS precisely target renal tubular epithelial cells in acute kidney injury (AKI), inhibiting the NLRP3 inflammasome and regulating the TGF-β pathway to reduce cisplatin-induced nephrotoxicity while avoiding burst-release oxidative stress [61,62]; in pancreatitis, cholesterol-modified ZIF-8 nanoparticles (loaded with CBS inhibitor AOAA) selectively lower pancreatic H_2_S (from 120 μM to 60 μM) to counter endogenous pro-inflammatory H_2_S, while PLGA-microencapsulated low-dose H_2_S (20 μM) activates PPAR-γ [[63], [64], [65], [66], [67]].

H_2_S's anti-inflammatory and immunomodulatory effects depend on the synergy of delivery systems (regulating release kinetics) and precise intervention in core signaling pathways. Future development should prioritize systems integrating inflammatory marker responsiveness and cell targeting to maximize H_2_S's therapeutic value.

### 3.2 Vasodilation and cardiovascular protection

H_2_S serves as a crucial regulator of vascular pathophysiology, coordinating hemodynamic homeostasis and lipid metabolism through a redox-sensitive signaling network [12,68]. Its mechanisms involve direct molecular interactions and pathway crosstalk, primarily inducing vasodilation by activating K_ATP_ channels on vascular smooth muscle cells—leading to membrane hyperpolarization and reduced Ca^2+^ influx—and exerting anti-atherosclerotic effects *via* synergistic PPAR-γ and Nrf2 pathway activation, along with upregulation of vascular endothelial growth factor (VEGF) to promote angiogenesis [[69], [70], [71], [72], [73], [74], [75]]. Although H_2_S exhibits concentration-dependent effects, its multi-target regulatory properties offer a strong basis for cardiovascular intervention strategies.

**Fig. 4:**
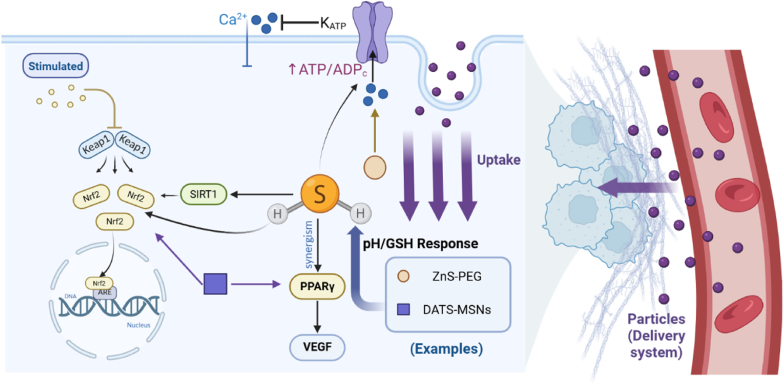
Multifunctional pathways of H_2_S in vasodilation and cardioprotection. (Figure was created with Biorender.com).

Nano-delivery systems offer significant therapeutic potential for hydrogen sulfide in vascular pathology (Fig. 4). For vasodilation, ZnS-PEG nanoparticles hydrolyze in ischemic acidic microenvironments (pH=6.0–6.5), releasing H_2_S to activate K_ATP_ channels and Zn^2+^ to enhance AKT-mediated endothelial migration, thereby alleviating cerebral ischemia/reperfusion injury [76]. Similarly, GSH/pH-dual-responsive DATS-loaded MIONs generate H_2_S specifically in the cytoplasmic GSH environment, preventing off-target vasodilation and maintaining myocardial perfusion [77].

In anti-atherosclerosis, H_2_S modulates lipid metabolism *via* PPAR-γ/Nrf2. DATS-loaded MSNs sustain H_2_S release, activating PPAR-γ/Nrf2 to reduce atherosclerotic plaque area in LDL receptor-deficient mice [78]. In addition to this, macrophage membrane-coated MM/ZnS/ATV nanoparticles target plaques *via* adhesion molecules: acidic microenvironment-triggered H_2_S inhibits foam cells, while co-delivered ATV lowers LDL, reducing plaque lipid core size in ApoE^−/−^ mice [56].

Other disease-oriented systems include: PLGA microspheres with protease-responsive ACS14 for inhaled treatment of pulmonary hypertension [79]; pH-sensitive ZIF-8-GYY4137 nanoparticles that release H_2_S in acidic inflammatory sites (pH ∼ 6.2) to inhibit aortic valve calcification [80]; and platelet membrane-camouflaged PM-MSN-DATS, which accumulates at cardiac injury sites *via* P-selectin, where sustained H_2_S release scavenges ROS and attenuates fibrosis, improving ventricular function in myocardial ischemia-reperfusion injury [81].

These examples confirm that “pathology-carrier matching” is essential for H_2_S efficacy. Future development should focus on delivery systems with multi-stimulus responsiveness and dual cellular targeting (e.g., endothelial and smooth muscle cells) to address complex cardiovascular diseases.

### 3.3 Anti-apoptotic and neuromodulation

**Fig. 5:**
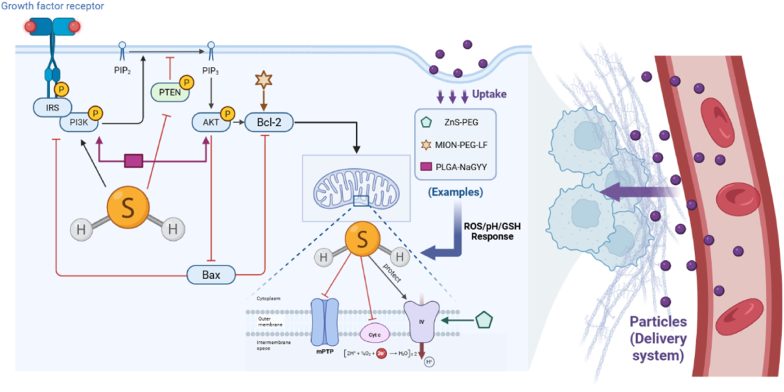
Neuroprotective mechanism of exogenously delivered hydrogen sulfide through modulation of the PI3K/AKT pathway and mitochondrial function. (Figure was created with Biorender.com).

Emerging evidence indicates that H_2_S is a crucial gaseous neuromodulator that confers concentration-dependent neuroprotection through complex regulation of the PI3K/AKT signaling pathway and mitochondrial function [38,[82], [83], [84], [85], [86], [87]]. Its key mechanisms involve S-sulfation of mitochondrial proteins to enhance ATP production and improve bioenergetic efficiency [[88], [89], [90], [91]]. At low concentrations, H_2_S protects mitochondrial complex IV activity, inhibits mitochondrial permeability transition pore (mPTP) opening, and reduces cytochrome C release, thereby blocking the mitochondrial apoptotic pathway [92,93]. Concurrently, it modulates the balance between pro-apoptotic and anti-apoptotic proteins by activating the PI3K/AKT signaling axis and inhibiting PTEN/Bax. Collectively, these actions enable H_2_S to coordinate the equilibrium between neuronal survival and cell death, forming the molecular basis for its therapeutic potential (Fig. 5).

The translation of H_2_S's neuroprotective effects relies on nanodelivery systems capable of crossing the blood-brain barrier (BBB) and achieving spatiotemporally controlled release to avoid neurotoxicity at high concentrations. ZnS-PEG nanoparticles hydrolyze in the acidic microenvironment of ischemic brain tissue (pH = 6.2–6.5), releasing H_2_S to preserve mitochondrial complex IV and Zn^2+^ to activate the AKT pathway, synergistically alleviating neuronal apoptosis in stroke models [76]. In Alzheimer's disease, PLGA-NaGYY microspheres maintain H_2_S within a physiological window (50–80 μM), activating PI3K/Akt to inactivate pro-apoptotic proteins and reduce β-amyloid-induced damage [94,95]. For Parkinson's disease, the BBB-penetrating MION-PEG-LF nanoparticle achieves cytoplasm-specific H_2_S release in the GSH-rich neuronal cytoplasm, thereby mitigating mitochondrial dysfunction without off-target effects [77].

In Parkinson's disease (PD), H_2_S mitigates mitochondrial dysfunction caused by CBS depletion [[96], [97], [98], [99], [100], [101]]. BBB-penetrating DATS@MION-PEG-LF nanoparticles (lactoferrin-modified) achieve cytoplasm-specific H_2_S release: under acidic endosomal conditions, DATS release is suppressed; in GSH-rich neuronal cytoplasm (5–10 mM), disulfide bond cleavage triggers H_2_S generation—this avoids off-target mitochondrial impairment and enhances ETC efficiency [77].

Other disease-specific applications further underscore the importance of “BBB penetration and pathological stimulus responsiveness” [102,103]. In traumatic brain injury, mesoporous silica nanoparticles delivering NaHS activate PI3K/Akt to suppress autophagy and reduce lesion volume [46].

These examples confirm that H_2_S's efficacy hinges on the synergy between advanced delivery systems and precise pathway regulation. Future development should focus on systems integrating dual BBB-targeting ligands and multi-stimulus responsiveness to address the complex pathophysiology of neurodegenerative diseases.

### 3.4 Dual roles in tumor promotion and suppression

The role of H_2_S in cancer biology is dichotomous, functioning as either a tumor promoter or suppressor depending on its concentration, which modulates redox homeostasis, metabolic reprogramming, and immune microenvironmental remodeling [104]. A central mechanism is its post-translational modification of proteins *via* S-persulfidation, which regulates key processes such as iron-dependent apoptosis (ferroptosis) and energy metabolism [105,106]. Metabolically, H_2_S can disrupt the Warburg effect by destabilizing hypoxia-inducible factor 1α (HIF-1α) and induce mitochondrial dysfunction, leading to impaired ATP synthesis. Paradoxically, the resulting intracellular acidification can synergize with chemotherapeutics to augment apoptosis, illustrating H_2_S's concentration-dependent duality.

**Fig. 6:**
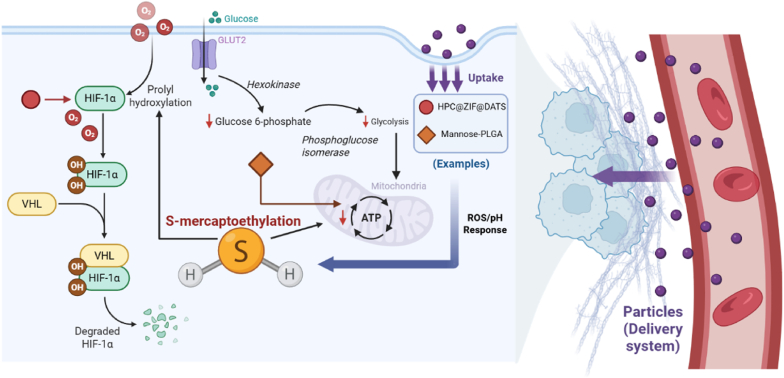
The tumor-killing effect of H_2_S *via* s-persulfation. (Figure was created with Biorender.com).

This bimodal effect is therapeutically exploitable through tumor microenvironment (TME)-responsive delivery systems, which provide spatiotemporal control to achieve high, cytotoxic H_2_S concentrations (>100 μM) at the tumor site while avoiding off-target promotion. As shown in Fig. 6, for instance, in anti-angiogenesis, acid-responsive HPC@ZIF@DATS nanoparticles degrade in TME (pH = 6.0–6.5) to release DATS, which generates H_2_S to downregulate HIF-1α target genes (VEGF, GLUT1). In breast cancer xenografts, this reduced microvessel density and tumor volume [107]. Conversely, low-dose GYY4137 delivered *via* hyaluronic acid (HA)-modified liposomes (targeting CD44^+^ cancer stem cells) maintains H_2_S at 30 μM, selectively activating Nrf2 to sensitize chemotherapy-resistant tumors to doxorubicin—enhancing apoptosis in colorectal cancer models [108].

In ferroptosis induction, H_2_S disrupts intracellular iron homeostasis by targeting glutathione peroxidase 4 (GPX4). FeS@BSA nanoparticles leverage TME-specific ROS to simultaneously release Fe^2+^ for Fenton reactions and H_2_S to inhibit GPX4, synergistically accumulating lipid peroxides and inducing ferroptosis in triple-negative breast cancer [109,110]. Mesoporous silica nanoparticles (MSNs) loaded with allyl isothiocyanate (AITC, an H_2_S donor) and Fe^3+^ achieve TME-responsive co-release: ATP-triggered pore opening releases AITC (generating H_2_S) and Fe^3+^ (reduced to Fe^2+^ by H_2_S), forming a “H_2_S–Fe^2+^” ferroptosis axis that shrank pancreatic tumors [111].

For immune modulation, H_2_S reprograms M2 tumor-associated macrophages (TAMs) to M1 phenotype *via* NF-κB activation [[112], [113], [114], [115], [116], [117], [118]]. Mannose-modified PLGA microspheres (targeting M2 TAM mannose receptors) release NaHS in response to ATP, with H_2_S upregulating pro-inflammatory cytokines (IL-1β, TNF-α) and reducing M2 markers (CD206) [119]. In glioblastoma, BBB-penetrating angiopep-2-modified ZIF-8 nanoparticles (ANG-ZIF-8) deliver GYY4137: pH-responsive H_2_S release (pH = 6.5) polarizes TAMs to M1, while concurrent VEGF inhibition reduces vascular permeability—improving checkpoint inhibitor efficacy [120].

H_2_S's tumor-regulating efficacy depends on “TME-responsive delivery systems” that tailor H_2_S concentration to pathological demands. Delivery systems must be engineered to achieve a sharp, high-concentration burst release exclusively within the tumor to trigger ferroptosis/apoptosis, while avoiding sub-threshold pro-tumoral concentrations. This requires platforms with robust tumor-targeting specificity (e.g., active targeting, EPR effect) and sharp stimulus-responsive release mechanisms [121].

## 4 Advanced H_2_S micro/nano delivery systems

### 4.1 Inorganic

#### 4.1.1 Metal sulfide nanomaterials

Metal sulfide nanomaterials have emerged as a promising inorganic platform for the controlled delivery of H_2_S, owing to their unique physicochemical properties, tunable morphologies, and responsive release mechanisms [122]. These systems leverage the inherent advantages of transition metal sulfides, such as high surface-to-volume ratios, redox activity, and compatibility with external stimuli (e.g., light, pH, or electric fields), to achieve precise H_2_S release in biomedical, industrial, and environmental applications.

Common metal sulfides include ZnS, MoS_2_, and CuS, whose nanostructures (e.g., nanosheets, nanomicrospheres, etc.) significantly improve the loading capacity and release kinetics of H_2_S. For instance, MoS_2_ nanosheets [123], which possess a substantial number of edge sites and defects, furnish active surfaces for the adsorption of H_2_S precursors or the catalytic generation of H_2_S *via* sulfur reduction reactions. Similarly, ZnS NPs (2–5 nm) prepared by the organic reverse micellar method can slowly release H_2_S for the treatment of ischemic stroke [76]. Furthermore, surface functionalization using targeted ligands, such as bone-targeting peptides, has been demonstrated to enhance the accumulation at specific sites and mitigate off-target effects [122].

**Fig. 7:**
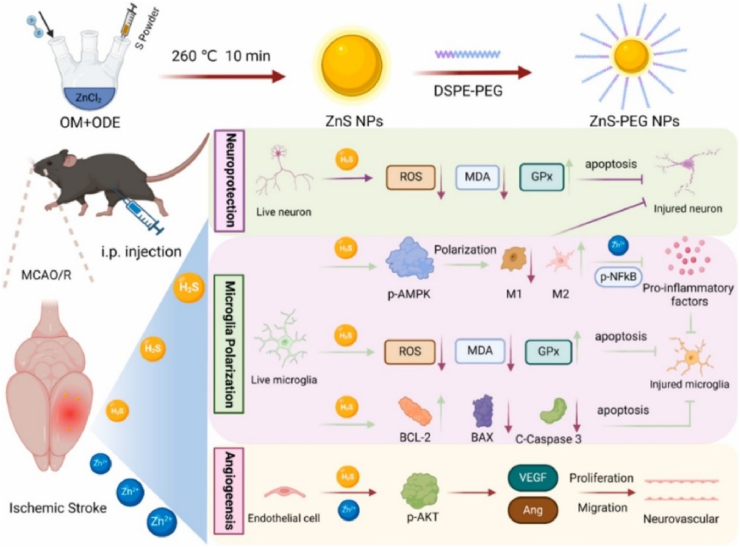
Synthesis of ZnS nanoparticles *via* an organic phase reverse method and subsequent DSPE-PEG modification to obtain ZnS-PEG NPs, which enable sustained H_2_S release *in vivo*. Evaluation using an ischemia/reperfusion model demonstrated the ability of ZnS-PEG NPs to suppress apoptotic pathways in multiple cell types, including neurons, microglia, and human brain microvascular endothelial cells (HBMECs) [76].

Release of H_2_S from metal sulfide systems triggered by pH stimulation: in acidic microenvironments (e.g., tumors or ischemic tissues), ZnS and FeS NPs catabolize and release both H_2_S and metal ions (Zn^2+^, Fe^2+^) to synergistically reduce oxidative stress and promote tissue repair [124]. This dual release mechanism not only provides H_2_S but also utilizes the properties of these metal ions (e.g., the antimicrobial properties of Zn^2+^ and Fe^2+^-triggered iron death) to exert a synergistic effect. ZnS-PEG NPs synthesized by Li et al. were stabilized by hydrolysis reaction for continuous release of H_2_S, inhibited neuronal apoptosis, and promoted vascular neogenesis in an acidic microenvironment (Fig. 7). Meanwhile, with the release of Zn^2+^, endothelial cell migration was enhanced by activating the AKT signaling pathway, which synergistically alleviated oxidative stress and inflammatory injury [76]. Similarly, FeS nanoparticles decompose in the microacidic environment of tumors, releasing H_2_S and Fe^2+^ while generating ROS *via* a Fenton-like reaction for a synergistic antitumor effect. Xie et al. [124] synthesized ferrous sulfide-embedded bovine serum albumin (FeS@BSA) nanoclusters by self-assembly, with H_2_S inhibiting peroxidases in tumors. H_2_S inhibited hydrogen peroxidase in the tumor and further promoted the Fenton reaction of Fe^2+^, which significantly facilitated the induction of intracellular ROS.

Advances in defect engineering and surface functionalization (e.g., doping with transition metals or grafting targeting ligands) will further optimize the loading efficiency and specificity of metal sulfide systems. Additionally, exploring biodegradable sulfides (e.g., FeS) could expand their use in eco-friendly applications. In summary, metal sulfide nanodelivery systems represent a versatile and robust platform for H_2_S delivery, with tunable release profiles tailored to diverse scenarios. Their integration with smart materials is expected to open a new field of precision gas therapy.

#### 4.1.2 Mesoporous nanomaterials

Mesoporous nanomaterials have gained considerable interest as advanced platforms for H_2_S delivery, owing to their exceptional structural versatility, high loading capacities, and compatibility with diverse functionalization strategies [125]. These systems effectively address key challenges in controlled H_2_S release, including premature leakage, low targeting efficiency, and environmental instability, by leveraging their tunable pore architectures, modifiable surface properties, and stimuli-responsive characteristics.

**Fig. 8:**
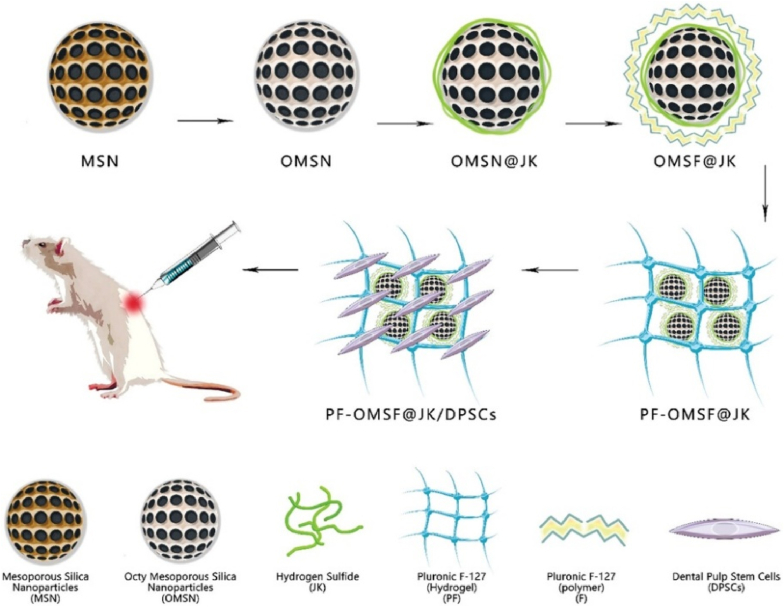
Schematic illustration of the preparation of an injectable thermosensitive hydrogel composite. Octyltriethoxysilane-functionalized mesoporous silica nanoparticles (OMSNs) loaded with the H_2_S donor JK-1 were modified with Pluronic F-127 (PF-127) and incorporated into a PF-127-based hydrogel matrix. This composite system enables the encapsulation of dental pulp-derived stem cells (DPSCs) with high viability, facilitating stem cell delivery while enabling sustained H_2_S release *in situ* [127]. Reprinted under the terms of the CC-BY.

Mesoporous silica nanoparticles (MSNs) offer an efficient platform for loading H_2_S donor molecules, owing to their ordered pore network (2–50 nm), high specific surface area (>500 m^2^/g), and readily modifiable surface chemistry [126]. For instance, Wang et al. [78] prepared DATS-loaded MSNs *via* a sol-gel method, demonstrating sustained H_2_S release over 3 h that delayed vascular endothelial apoptosis and attenuated graft ischemia–reperfusion injury. MSNs can also be integrated with other functional materials to enhance therapeutic outcomes. For example, Peng et al. [127] incorporated MSNs loaded with the H_2_S donor JK into a thermosensitive hydrogel, which promoted macrophage M2 polarization and stem cell differentiation *in vitro*, and significantly improved recovery in a rat spinal cord injury model. In such systems, MSNs act as nanoscale depots for H_2_S storage and controlled release **(**Fig. 8**)**.

**Fig. 9:**
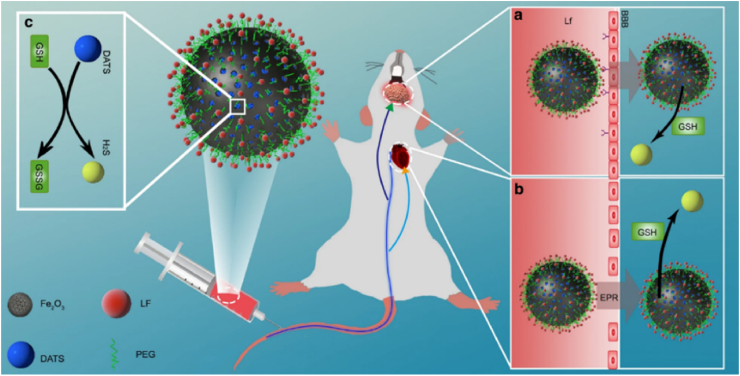
Schematic illustration of a DATS-loaded MION nanoparticle functionalized with PEG and LF to achieve prolonged circulation and brain-targeting capabilities. The system enables GSH-triggered slow release of H_2_S in target tissues [77]. Reprinted under the terms of the CC-BY 4.0.

Beyond MSNs, mesoporous iron oxide (MION) has emerged as a promising alternative for mesoporous delivery systems [128]. In contrast to conventional silica-based carriers, MIONs integrate strong chemical stability, biocompatibility, and structural versatility with intrinsic magnetic properties, rendering them especially suitable for H_2_S delivery applications that demand targeted transport, environmental responsiveness, and synergistic therapy [129]. A representative example is the DATS@MION-PEG-LF system developed by Sun et al., which employs a sophisticated dual-triggered release mechanism. The core process involves GSH-triggered H_2_S release from the encapsulated DATS donor. In the cytoplasmic GSH environment, the disulfide bond in DATS undergoes cleavage *via* nucleophilic attack by glutathione thiol groups, initiating a series of thiol-exchange reactions that ultimately generate H_2_S. Crucially, this GSH-mediated process is strategically modulated by the MION carrier. Under acidic conditions (e.g., within endosomes), DATS release is suppressed, thereby inhibiting H_2_S production in non-target compartments. This design ensures that H_2_S generation is preferentially activated in the cytoplasm of target cells with elevated glutathione levels, minimizing premature leakage and enhancing therapeutic precision (Fig. 9). Unlike MSNs, MIONs can be fully metabolized and are readily traceable *in vivo*. Moreover, while MSNs are prone to plasma protein adsorption, exhibit limited circulation time, and cannot cross the BBB, restricting their utility in the central nervous system (CNS), MIONs offer distinct advantages in these aspects. The dual-triggered mechanism of DATS@MION-PEG-LF enables sustained H_2_S release, a critical feature for alleviating myocardial ischemia–reperfusion injury [77].

Surface engineering of MIONs enhances their therapeutic precision. PEG coating reduces opsonization, prolonging circulation time, while lactoferrin (LF) modification enables BBB penetration for neuroprotection [130]. In a cardiac arrest/cardiopulmonary resuscitation (CA/CPR) model, LF-functionalized MIONs demonstrated dual targeting to both brain and heart tissues, reducing neuronal apoptosis by 40 % and myocardial infarct size by 50 % through H_2_S-mediated suppression of oxidative stress markers (e.g., malondialdehyde) and enhancement of superoxide dismutase activity [77]. Furthermore, the inherent magnetism of iron oxide allows external magnetic field-guided accumulation, a feature exploited in tumor-targeted therapies where localized H_2_S release synergizes with photothermal or chemodynamic effects.

Despite these advances in mesoporous materials, challenges remain. Long-term biosafety concerns, such as oxidative stress induced by silica and metal ions (e.g., Zn^2+^, Fe^2+^) accumulation, need to be further validated by degradation kinetic studies and chelator-assisted scavenging strategies.

### 4.2 Organic

#### 4.2.1 Polymers

Polymer-based carriers have emerged as a key strategy for the controlled and targeted delivery of H_2_S, offering more tunable structural and chemical flexibility and better biocompatibility than inorganic delivery systems [131]. Polymeric carriers include polymer nanoparticles and polymeric micelles. Polymeric nanoparticles, such as poly(lactic-hydroxyacetic acid) copolymer (PLGA) (or poly(ε-caprolactone) (PCL)-based carriers, are capable of sustained release of H_2_S [132]. For example, PLGA microspheres loaded with the ACS14 have been engineered for pulmonary delivery to achieve prolonged H_2_S release for the treatment of pulmonary hypertension [79]. Recent studies have emphasized the effect of polymer morphology on the efficiency of H_2_S release. Tubular polymer bodies formed by membrane stretching of sulfur-rich block copolymers exhibited enhanced cysteine-triggered H_2_S release compared to spherical polymers [133]. The elongated membrane geometry contributes to faster gas diffusion and improves anticancer effects in tumor models.

Polymeric micelles are usually formed by self-assembly from amphiphilic block copolymers (e.g., PEG-b-PCL) with a hydrophobic core and hydrophilic shell structure. Usually, the precursor drug of H_2_S is encapsulated in the hydrophobic core, and the controlled release is carried out using a cross-linking strategy designed by chemical bonding [134]. The nuclear cross-linked micelles (CCM) developed by Rong et al. [135] achieved simultaneous cross-linking and coupling with the H_2_S donor at the core of the micelles by using p-disubstituted S-arylthiooximes (*p*-diSATOs) as the cross-linking agent. The design initially achieved rapid release of H_2_S to promote wound healing through electron-withdrawal of *p*-diSATOs, and subsequently maintained the slow release of H_2_S through the stability of the crosslinked structure, which ultimately resulted in a 40 % increase in wound healing efficiency in a mouse burn model. In addition, polymeric micelles containing ADT were designed by Jerry J. Y. Chen et al. [136]. These micelles can modulate the rate of H_2_S release by self-assembling to form two different morphologies, spherical and worm-like. The ADT groups within the micelles can be oxidized by intracellular ROS to release H_2_S. It was found that the thermodynamic stability of the micelles can be controlled by adjusting the design of the polymers to regulate the rate of H_2_S release. The more stable the micelles, the slower the rate of H_2_S release, and the better tumor inhibition *in vivo* and *in vitro*.

**Fig. 10:**
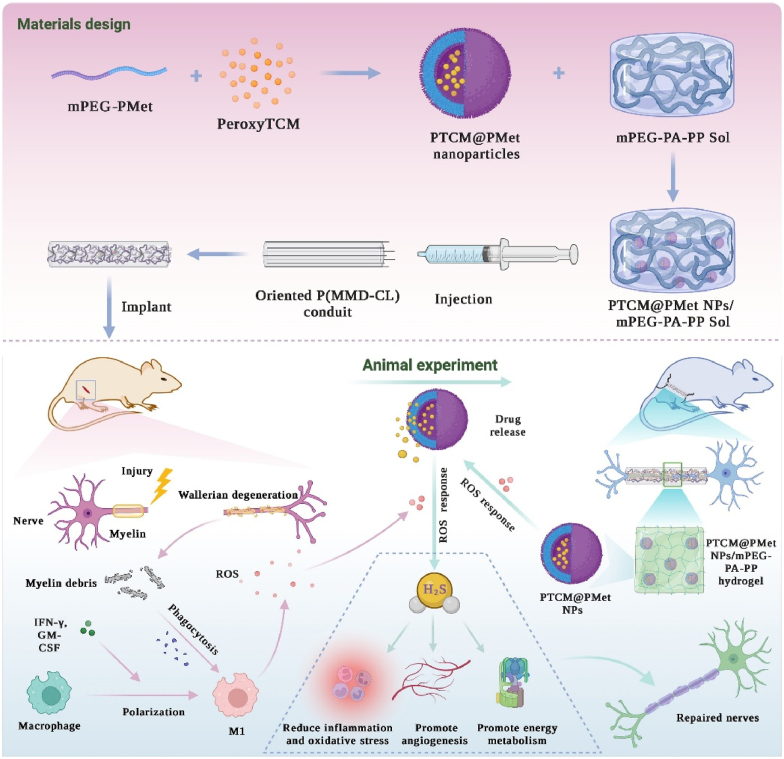
Preparation of a bionic neural scaffold *via* ROS-responsive mPEG-PMet polymer assembly loaded with Peroxy-TCM, incorporation into a thermosensitive hydrogel, and injection into a P(MMD-CL) catheter. Upon ROS triggering *in vivo*, the scaffold releases H_2_S, polarizing macrophages toward the M2 phenotype, attenuating local inflammation and oxidative stress, and promoting angiogenesis and energy metabolism at the injury site [54].

Polymeric nanoparticles are also often used in combination with other carriers to achieve differentiated and efficient drug delivery targeting the pathological microenvironment. Dong et al. [54] constructed a slow-release H_2_S neural graft by integrating a polymer (mPEG-PMet) containing a H_2_S donor (peroxythiocarbamate, PeroxyTCM) into a temperature-sensitive hydrogel (mPEG-PA-PP) and injecting it into an electrostatically rotating catheter [P(MMD-CL)] (Fig. 10). After implantation of this system, ROS generated at the site of injury prompted mPEG-PMet to release PeroxyTCM, and H_2_S generated after a series of reactions regulated macrophage polarization toward the M2 phenotype, reduced inflammatory inflammatory factor expression, and ultimately achieved efficient repair of nerve injury.

Key future directions in polymer carriers include: integration of active targeting ligands (e.g., peptides, antibodies) can improve specificity for diseased tissues; and exploring more non-spherical polymers to enhance gas diffusion and therapeutic efficacy.

#### 4.2.2 Liposomes

Lipid-based carriers, especially liposomes, have become the cornerstone of controlled and targeted delivery of H_2_S because of their excellent biosafety and bioavailability [118]. Liposomes are self-assembled spherical vesicles composed of phospholipid bilayers that offer unrivaled versatility in encapsulating hydrophilic and hydrophobic H_2_S donors. Their structural flexibility allows for precision engineering of stimuli-responsive release, extended circulation, and tissue-specific targeting more readily than other carriers [11].

Liposomes encapsulate H_2_S donors through three primary mechanisms: (1) hydrophobic core loading, where slow-releasing donors like DATS or GYY4137 are embedded within the lipid bilayer [137]; (2) aqueous core loading, which traps water-soluble donors such as NaHS or Na_2_S in the hydrophilic lumen, often stabilized by pH-gradient techniques to prevent premature burst release [138]; and (3) membrane-anchored donors, where thiol-responsive moieties like S-aroylthiooxime (SATO) are conjugated to lipid headgroups through cleavable disulfide bonds [11].

Surface modification further enhances liposome performance. Polyethylene glycolization prolonged the circulating half-life from 2-4 h to 24–48 h by reducing the conditioning action and macrophage uptake [139]. For example, Chiwoo et al. [140] tested various polyethylene glycol ratios to develop GYY4137-containing liposomes with high stability and efficient spleen-targeting ability and validated their therapeutic potential in a dextran sodium sulfate (DSS)-induced colitis model. However, in this experiment, it was also found that a decrease in H_2_S release was accompanied by an increase in the composition of the PEG and a prolongation of the *in vivo* circulation time. This is a result of reduced cellular uptake due to the stealth properties of PEG and needs to be addressed by utilizing other strategies, such as pH-sensitive lipids or in combination with sonication.

pH-sensitive lipids (e.g., DOPE) protonate their carboxylic acid groups under acidic conditions in tumors, resulting in the transformation of the lipid bilayer from a stable lamellar structure to a non-lamellar hexagonal crystalline phase (H11 phase), which destabilizes the liposome membrane [141]. There are also some liposomes with surface-modified polyelectrolytes (e.g., PEAA), and conformational changes in the polyelectrolyte chains in acidic environments can trigger liposome rearrangements and accelerate drug release. Currently, fewer studies have been conducted on the use of pH-sensitive liposomes for loading H_2_S donors. In the future, the reduced membrane stability in the tumor microenvironment can be exploited to achieve regional release of H_2_S in tumor therapy, thus precisely controlling the H_2_S concentration at the lesion site.

**Fig. 11:**
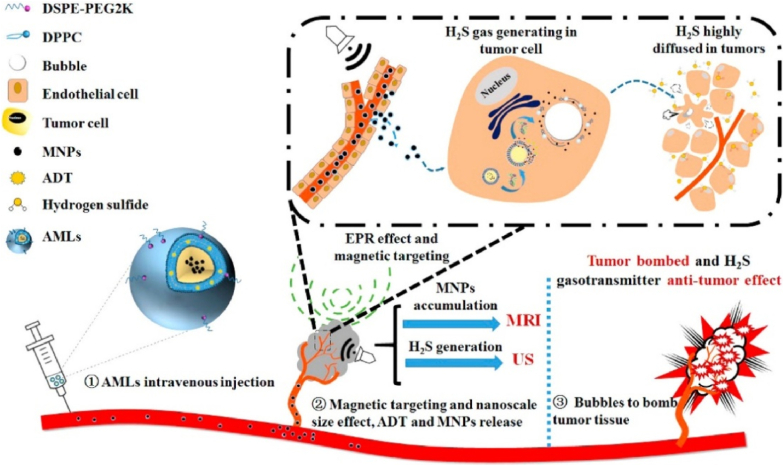
Magnetic targeting liposomes assembled from ADT and superparamagnetic nanoparticles enable tumor-specific accumulation under external magnetic guidance. These liposomes generate *in situ* microbubbles that serve as contrast agents for MR/US dual-modal imaging, while also achieving effective tumor cell ablation through acoustic cavitation at higher ultrasound intensities [107].

When combined with other strategies such as ultrasound, liposomes enable region-specific drug delivery. Liu et al. [107] developed unique stimulus-responsive magnetic liposomes encapsulating ADT, which are targeted by an external magnetic field to reach a specific tumor site. ADT generates H_2_S microbubbles, which can be monitored multimodally by ultrasound imaging and magnetic resonance imaging (Fig. 11). In addition, under high-intensity ultrasound stimulation, H_2_S microbubbles explode inside the tumor to physically kill the tumor, and then H_2_S acts as a gas carrier to diffuse deeper into the tumor, exerting its chemotaxis and ultimately showing higher anticancer efficiency.

### 4.3 Metal-organic frameworks (MOFs)

Organic-inorganic hybrid materials, especially MOFs, enable precise H_2_S loading, targeted delivery, and stimulus-responsive release due to their tunable porosity and programmable functionality [108,142].

MOFs, composed of metal ions/clusters and organic linkers, have been extensively explored for H_2_S delivery due to their structural versatility and redox-active sites. The limited specific surface area of conventional adsorbents imposes constraints on H_2_S adsorption capacity, which has driven current research efforts toward developing MOFs with enhanced H_2_S adsorption capacity and selectivity. For instance, the coupling between Ni_3_(HITP)_2_ and NUS-8 nanosheets creates synergistic active sites, enabling ultrasensitive H_2_S detection at parts-per-billion (ppb) levels (LOD: ∼6 ppb) [142]. Notably, the reversible adsorption-desorption characteristics of MOFs enable their potential application as precision-controlled H_2_S delivery systems, particularly for achieving spatiotemporal regulation of endogenous H_2_S release in physiological environments. Chen et al. utilized the properties of MOF as a hydrogen bond donor and weak hydrogen bond acceptor to achieve reversible adsorption of H_2_S using Zr_6_O_4_(OH)_4_ and MOF-801 framework [143].

Similarly, Wang et al. [144] developed a hyaluronic acid (HA)-, PEG-, and cholesterol (CLS)-functionalized zeolitic imidazolate framework (ZIF) loaded with DATS, designated as HPC@ZIF@DATS, for therapeutic intervention in hindlimb ischemia. This nanotherapeutic system employs a dual-targeting strategy: Upon intramuscular injection into the gastrocnemius, HA-mediated CD44 receptor binding facilitates selective nanoparticle internalization by macrophages. The release process is initiated in the acidic compartments of the endosome/lysosome, where the imidazole-based ligands become protonated, leading to the dissolution of the pH-responsive ZIF-8 framework. This structural breakdown releases the encapsulated DATS into the cytoplasm. Within the reducing cytoplasmic environment rich in GSH, the disulfide bonds in DATS undergo nucleophilic attack by the thiol anion, resulting in the controlled generation of H_2_S. This enzyme-independent mechanism ensures direct and efficient H_2_S production inside target cells, facilitating downstream biological responses such as promoting macrophage polarization toward the reparative M2 phenotype. Concurrently, zinc ions liberated from ZIF-8 degradation activate endothelial morphogenetic pathways. Impressively, HPC@ZIF@DATS demonstrates synergistic therapeutic effects through two complementary mechanisms: (1) Zinc-H_2_S crosstalk amplifies angiogenic signaling by VEGF upregulation, and (2) H_2_S-mediated scavenging of ROS coupled with anti-inflammatory cytokine modulation establishes a regenerative microenvironment. Both *in vitro* and *in vivo* evaluations confirm enhanced neovascularization and functional recovery in ischemic tissues.

### 4.4 Cell membrane-coated nanoparticles (CMNPs)

CMNPs represent a breakthrough in targeted H_2_S delivery, synergizing biointerface engineering with controlled therapeutic release. These nanovehicles utilize native membrane components to simultaneously address three critical challenges in drug delivery: 1) Biocompatibility enhancement through surface marker preservation, 2) Immune evasion through CD47-SIRPα checkpoint signaling, and 3) Tissue-specific targeting mediated by membrane protein recognition [80]. The core-shell architecture combines a H_2_S-prodrug-loaded synthetic matrix with biologically functionalized membranes sourced from erythrocytes, platelets, or tumor cells. Key mechanistic advantages emerge from this design: Erythrocyte-derived membranes exploit CD47's “don't eat me” signaling to reduce macrophage clearance (3-fold circulation time extension vs conventional nanoparticles), while tumor cell membranes enable homologous targeting through integrin-mediated cellular recognition [145]. The system capitalizes on pathophysiological gradients through a dual-phase targeting mechanism—initial passive accumulation *via* enhanced vascular permeability, followed by active uptake mediated by membrane protein interactions [110].

Blood cell membrane-derived carriers, particularly those derived from platelets and erythrocytes, have gained significant attention in vascular therapeutics due to their innate biocompatibility and disease-targeting capabilities for H_2_S delivery. Platelet membranes are uniquely advantageous in vascular applications owing to their surface expression of receptors such as P-selectin, which enables active recognition and binding to inflammation sites and damaged endothelial cells [111]. This targeting mechanism ensures localized H_2_S delivery while minimizing systemic exposure. In contrast, erythrocyte membranes exhibit exceptional deformability, allowing them to navigate through stenotic vessels and accumulate in ischemic regions, thereby enhancing therapeutic efficacy in hypoxic microenvironments [146].

A notable example is the work by Chen et al. [81], who engineered platelet membrane-camphorated mesoporous silica nanoparticles (PM-MSN-DATS) for myocardial ischemia-reperfusion injury (MIRI) treatment. These nanoparticles encapsulated DATS, a slow-release H_2_S donor, within a platelet membrane shell. *In vivo* studies demonstrated that PM-MSN-DATS selectively accumulated at cardiac endothelial injury sites in MIRI rats, where sustained H_2_S release effectively scavenged ROS, reduced myocardial fibrosis, and improved cardiac function. The platelet membrane's inherent affinity for vascular damage sites synergized with H_2_S's cytoprotective effects, highlighting the dual advantages of biological targeting and gas-mediated therapy.

In another study targeting MIRI, Liu et al. [119] coloaded platelet membrane vesicles with rapamycin, a mTOR inhibitor, and JK-1, a H_2_S donor that triggers responses to inflammation. This system achieved sequential drug release in hypoxic/reoxygenated cardiomyocytes: first, the mTOR pathway inhibition mitigated apoptosis, while the inflammatory microenvironment-triggered H_2_S release polarized macrophages toward an anti-inflammatory M2 phenotype. The combined effects significantly attenuated myocardial injury and promoted tissue repair, underscoring the potential of membrane-based carriers to integrate gasotransmitter delivery with conventional pharmacotherapy for enhanced outcomes.

**Fig. 12:**
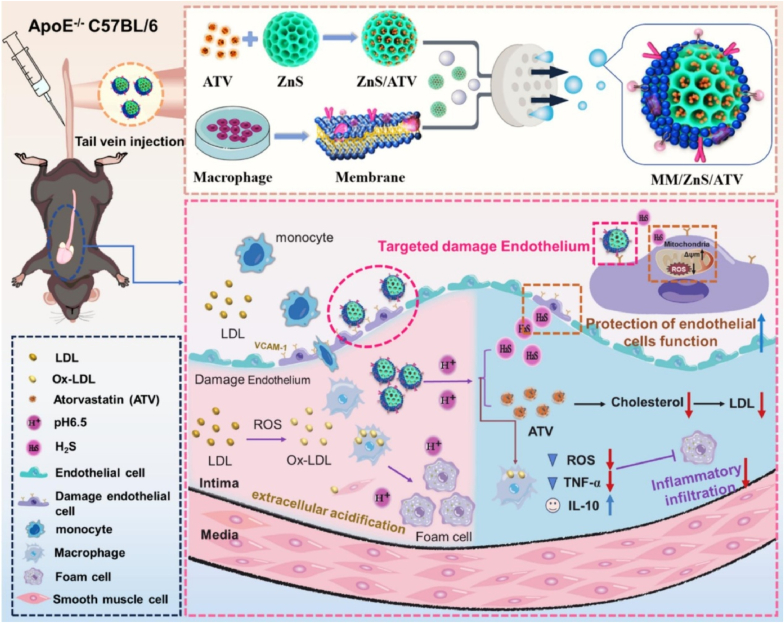
An MM/ZnS/ATV nanoplatform for atherosclerosis treatment. The MM carrier targets injured endothelial cells and encapsulates ZnS particles loaded with atorvastatin (ATV). Following cellular uptake, the acidic environment triggers simultaneous release of H_2_S from ZnS hydrolysis and ATV, which collectively improve lipid metabolism, suppress inflammatory factor expression, and promote endothelial repair [56].

Macrophage membranes (MM) exhibit superior advantages over blood cell membranes in drug delivery systems, particularly through their enhanced capacity for active tissue penetration and immunomodulation within complex pathological microenvironments [120]. These properties stem from their inherent expression of chemokine receptors and adhesion molecules, enabling precise targeting of inflamed or diseased tissues. A pioneering example is the multi-targeted, pH-responsive nanoplatform (MM/ZnS/ATV) developed by Xie et al. [56] for atherosclerosis therapy. This system utilizes MM coatings to achieve lesion-specific targeting of atherosclerotic plaques, capitalizing on the membrane's natural affinity for inflammatory vascular sites. The nanoplatform's design integrates ZnS as a H_2_S-generating component and atorvastatin (ATV) as an anti-inflammatory agent, synergistically addressing multiple pathological pathways. The MM/ZnS/ATV system demonstrates pH-dependent H_2_S release kinetics, with a cumulative concentration of 12.3 μM over 24 h under acidic conditions (pH = 6.5, mimicking atherosclerotic plaques), compared to only 3.6 μM in neutral environments (pH = 7.4). This acid-triggered release mechanism ensures spatially controlled H_2_S delivery, which directly correlates with therapeutic outcomes. *In vitro* studies revealed that the released H_2_S significantly suppresses pro-inflammatory cytokine TNF-α while upregulating anti-inflammatory IL-10 expression, effectively polarizing macrophages toward a reparative M2 phenotype. *In vitro* and *in vivo* studies confirmed the favorable therapeutic effects of the MM/ZnS/ATV nanoplatform, showing its potential for the treatment of atherosclerosis (Fig. 12).

### 4.5 Compatibility between H_2_S donors and delivery platforms

The preceding sections have detailed various sophisticated nanoplatforms for H_2_S delivery. As noted at the end of section 2, the performance of these systems depends not only on the carrier design but also critically on the choice of H_2_S donor. The interplay between the physicochemical properties of the donor and the material characteristics of the carrier constitutes a fundamental, yet frequently underestimated design consideration, governing release kinetics, targeting precision, and ultimately therapeutic efficacy. A thorough understanding of donor–carrier compatibility is therefore essential for developing systems that function synergistically, rather than merely additively.

An ideal pairing maximizes the strengths of both components. For example, hydrophobic donors such as DATS or ADT are inherently suitable for encapsulation within the lipid bilayer of liposomes or the hydrophobic core of polymeric microcapsules, as their compatibility ensures high loading efficiency and enhanced stability during circulation. Conversely, hydrophilic donors like NaHS or GYY4137 can be efficiently loaded into the aqueous interior of liposomes or the mesoporous channels of MSNs/MOFs. Furthermore, the integration of stimulus-responsive donors (e.g., ROS-responsive PeroxyTCM, pH-responsive JK series) into equally responsive nanomaterials (e.g., pH-degradable ZIF-8, ROS-sensitive polymers) establishes a dual-control mechanism. This synergistic design ensures that H_2_S release is precisely confined to the pathological microenvironment, thereby minimizing off-target effects and improving therapeutic precision. A summary of rational donor-platform pairing strategies categorized by donor properties, is presented in Table 2.

**Table 2:** Rational design of H_2_S donor and delivery system pairs.

Typical H_2_S donors	Physical and chemical properties	Adaptive delivery system	Example of synergistic effect	Potential antagonistic effect
Inorganic donor (NaHS, Na_2_S)	High water solubility; easily burst release	Metal sulfide nanomaterials; liposomes	ZnS-PEG NPs loaded with NaHS: synergistic anti-inflammatory effects of Zn^2+^ and H_2_S [76].	Combination with hydrophobic polymers: PEG modification is required to improve compatibility
Organic slow-release donor (GYY4137, DATS)	Highly hydrophobic; slow-release	MOFs; Polymers	HPC@ZIF@DATS: H_2_S release enhanced in acidic ischemic condition [144].	Combination with hydrophilic liposomes: Prone to aggregation, requiring surface modification to enhance stability.
Stimulus-responsive donor (PeroxyTCM, JK-2)	ROS/pH-dependent release	Smart Response Polymers	mPEG-PMet loaded with PeroxyTCM: ROS-triggered H_2_S release efficiency at the site of nerve injury increases [54].	Combination with non-responsive carriers: Fails to trigger targeted release, leading to increased off-target toxicity.

While synergistic effects are achievable, inappropriate donor–carrier combinations can result in functional impairment. For example, encapsulating a highly hydrolyzable donor in a slowly degrading polymer matrix may lead to premature carrier breakdown or abrupt release due to accumulated gaseous pressure. Similarly, the intrinsic chemical instability of certain donors under specific fabrication conditions such as during high-temperature synthesis of metal sulfide nanoparticles must be carefully evaluated. Therefore, the selection of a delivery platform should consider not only the pathological context but also key donor properties, including chemical stability, reactivity, and release kinetics.

In summary, the intentional molecular and material-level design of donor-delivery systems that move beyond simple physical encapsulation is paramount. Future efforts should focus on systematically evaluating donor–carrier compatibility, establishing clear structure-activity relationships, and developing novel donors specifically engineered for integration with advanced nanocarriers. Such rational design is crucial to fully realize the potential of precision H_2_S therapy.

### 4.6 Comparative analysis of micro/nano-delivery systems

The discussion on donor-delivery system compatibility underscores the critical importance of selecting the appropriate nanocarrier. As detailed in sections 4.1 to 4.4, each type of nanoplatform possesses unique intrinsic characteristics. To facilitate a comprehensive understanding and guide the selection process based on specific application requirements, a comparative analysis of their key features is essential. Table 3 provides a systematic overview of the advantages, limitations, and primary applications of the major nanodelivery platforms discussed in this review.

**Table 3:** Comparative summary of advanced H_2_S micro/nano-delivery system.

Delivery System	Key Advantages	Major Challenges	Ideal Application Scenarios
Metal sulfide nanomaterials	High stability; Controllable synthesis; Multi-modal functionality (e.g., imaging, synergistic therapy)	Potential long-term metal toxicity; Relatively low loading for exogenous donors.	Tissue repair; Synergistic therapy (e.g., chemodynamic); Theranostics.
Mesoporous nanomaterials	Well-defined porosity; Tunable surface chemistry; Good biocompatibility	Poor biodegradability; Potential inflammatory response; Premature leakage risk.	Controlled release; Co-delivery systems; Stimuli-responsive delivery.
Polymers	Excellent biocompatibility and biodegradability; Tunable mechanical properties	Complexity in synthesis; Potential initial burst release; Low hydrophilic loading.	Sustained release; Hydrophobic donor delivery; Injectable depots.
Liposomes	High biocompatibility; Co-delivery capacity; Clinically translated technology	Stability issues (storage); Rapid clearance without PEGylation; Leakage.	Versatile delivery; Triggered release; Clinical translation.
MOFs	Extremely high surface area; Designable functionality; Tunable porosity.	Stability in biological media; Potential toxicity of ligands/metals; Costly synthesis.	Ultra-high payload delivery; Sensitive detection; Gated release.
CMNPs	Long circulation; Immune evasion; Specific targeting to source cells' pathological sites.	Complex preparation; Batch-to-batch variability; Source of cell membranes.	Targeted delivery; Complex therapies; Immune modulation.

As summarized in Table 3, the landscape of H_2_S micro/nano delivery systems presents a series of trade-offs: the pursuit of an “ideal” nanoplatform involves balancing a triple challenge—precision, efficacy, and safety. No single platform is absolutely superior; each occupies a unique position defined by compromises between its inherent properties, such as manufacturing complexity, drug loading capacity, targeting accuracy, and biosafety. For instance, while CMNPs offer unparalleled targeting and stealth capabilities, their intricate manufacturing process poses scalability challenges. Conversely, polymeric nanoparticles exhibit excellent biocompatibility and controllable release properties but typically require additional modifications to achieve effective targeting.

This comparative analysis indicates that the future of precise H_2_S delivery likely lies not in pursuing a single ideal material, but in the judicious combination and integration of these platforms. Integration strategies, such as encapsulating metal sulfide nanoparticles within biodegradable polymers and further coating their surfaces with cell membranes, aim to synergize the strengths of each component while mitigating their respective limitations. The ultimate goal is to fabricate intelligent, multi-layered nanodevices capable of seamlessly traversing biological barriers, responding to specific disease stimuli, and releasing H_2_S with precise spatiotemporal control—thereby safely unlocking its full therapeutic potential.

## 5 Conclusions and future directions

H_2_S, once regarded merely as a toxic gas, has emerged as a critical gaseous signaling molecule with profound therapeutic potential across diverse diseases. This review emphasizes the clinical potential of H_2_S-related therapies, based on the different responses of H_2_S to different pathophysiological conditions.

Traditional H_2_S donors, including inorganic salts and slow-release compounds, have played an important role in revealing the physiological functions of gas transmitters. However, their clinical translation is hampered by limitations such as burst release kinetics, poor tissue specificity, and dose-dependent toxicity. For example, sulfide salts produce transient H_2_S spikes that may trigger oxidative stress, whereas synthetic donors such as GYY4137 lack spatial control in complex disease microenvironments despite longer release times. Therefore, donors based on pH, protease expression profile, ROS, and other environmental responses were developed with programmable molecular structures that support fine-tuning of release rates and targeting.

Therapeutic applications of H_2_S are multifaceted. Its anti-inflammatory and immunomodulatory effects mediated by NF-κB peroxidation and promotion of macrophage conversion to the M2 type show potential in COPD, AKI, and pancreatitis. In cardiovascular diseases, H_2_S-promoted Nrf2 nuclear translocation in concert with PPARγ and K_ATP_ channels ameliorates atherosclerosis and hypertension. In neurodegenerative diseases, H_2_S acts on the PI3K/Akt pathway and mitochondria, thereby attenuating apoptosis and oxidative stress, with significant therapeutic effects in AD and PD, but requires precise spatiotemporal delivery to avoid off-target neurotoxicity. This duality extends to tumors, where H_2_S exemplifies low concentration promotion and high concentration inhibition, making the development of spatiotemporally controlled and tissue-specific carriers imminent.

Based on the above, advanced micro/nano drug delivery platforms aimed at overcoming pharmacokinetic barriers have emerged. Innovations include organic carriers using inorganic materials, including metal sulfide nanomaterials and mesoporous nanoparticles, organic carriers such as polymers or lipids for sustained delivery, and CMNPs with good biocompatibility utilizing natural targeting mechanisms, etc. MOFs further exemplify the fusion of high loading capacity and stimulus-triggering precision, offering promising avenues for clinical therapy.

However, challenges remain, and the biphasic “concentration-effect” relationship of H_2_S requires more stringent dose control, especially in cancer therapy, and recent advances in H_2_S-encapsulated microbubble/nanobubble systems offer promising solutions to this challenge. Specifically, air-core bubbles with functional shells exhibit three unique advantages: (1) acoustic cavitation-triggered ultrasound-responsive burst release enables spatiotemporal precision; (2) the air-liquid interface facilitates multimodal integration, allowing for the simultaneous monitoring of H_2_S release *via* photoacoustic imaging or MRI tracking (3) the hierarchical structure (e.g., re-hybridized cell membranes); enhances tissue targeting.

Meanwhile, as new physiological functions of H_2_S continue to be discovered, exploring subcellular-level H_2_S kinetics becomes critical and requires quantifying the differences in the specific mechanisms of action by which different donors produce different concentrations of H_2_S. Ultimately, the utilization of H_2_S biology through interdisciplinary collaborations may open up new paradigms in precision medicine.

## CRediT authorship contribution statement

**Huiting Xu:** Conceptualization, Resources, Software, Validation, Writing – original draft, Writing – review & editing. **Yang Liu:** Funding acquisition, Resources. **Tiandong Chen:** Validation. **Mingxi Li:** Resources. **Fang Yang:** Funding acquisition, Resources, Supervision, Validation, Writing – original draft, Writing – review & editing.

## Declaration of competing interest

The authors declare that they have no known competing financial interests or personal relationships that could have appeared to influence the work reported in this paper.

## Data Availability

Data will be made available on request.
